# Neuregulin-4 Is Required for Maintaining Soma Size of Pyramidal Neurons in the Motor Cortex

**DOI:** 10.1523/ENEURO.0288-20.2021

**Published:** 2021-02-23

**Authors:** Blanca Paramo, Sven O. Bachmann, Stéphane J. Baudouin, Isabel Martinez-Garay, Alun M. Davies

**Affiliations:** School of Biosciences, Cardiff University, Cardiff CF10 3AX, Wales

**Keywords:** cortical development, neuregulin-4, neuronal soma size, pyramidal neurons

## Abstract

The regulation of neuronal soma size is essential for appropriate brain circuit function and its dysregulation is associated with several neurodevelopmental disorders. A defect in the dendritic growth and elaboration of motor neocortical pyramidal neurons in neonates lacking neuregulin-4 (NRG4) has previously been reported. In this study, we investigated whether the loss of NRG4 causes further morphologic defects that are specific to these neurons. We analyzed the soma size of pyramidal neurons of layer (L)2/3 and L5 of the motor cortex and a subpopulation of multipolar interneurons in this neocortical region in *Nrg4*^+/+^ and *Nrg4*^−/−^ mice. There were significant decreases in pyramidal neuron soma size in *Nrg4*^−/−^ mice compared with *Nrg4*^+/+^ littermates at all stages studied [postnatal day (P)10, P30, and P60]. The reduction was especially marked at P10 and in L5 pyramidal neurons. Soma size was not significantly different for multipolar interneurons at any age. This *in vivo* phenotype was replicated in pyramidal neurons cultured from *Nrg4*^−/−^ mice and was rescued by NRG treatment. Analysis of a public single-cell RNA sequencing repository revealed discrete *Nrg4* and *Erbb4* expression in subpopulations of L5 pyramidal neurons, suggesting that the observed defects were due in part to loss of autocrine Nrg4/ErbB4 signaling. The pyramidal phenotype in the motor cortex of *Nrg4*^−/−^ mice was associated with a lack of Rotarod test improvement in P60 mice, suggesting that absence of NRG4 causes alterations in motor performance.

## Significance Statement

Neuregulins are growth factors that are abundantly expressed in the nervous system where they regulate a plethora of processes essential for normal nervous system development and function in adulthood. Dysregulation of neuregulin signaling has been implicated in neurodevelopmental disorders, thus characterizing the particular functions of members of this family of proteins is highly relevant for understanding how such disorders emerge. This study shows that neuregulin-4 (NRG4) is required to maintain motor cortex pyramidal neuron soma size, and that altered pyramidal neuronal morphology is associated with motor defects in mice.

## Introduction

Neuregulins are signaling proteins that are abundantly expressed in the nervous system where they are required for neuronal development and brain function. Since the discovery of the first member of this family of proteins [neuregulin-1 (NRG1)], five other members have been described, each one with multiple isoforms generated by alterative splicing (Aono et al., 2000; Kanemoto et al., 2001; Hayes and Gullick, 2008). Neuregulins bind and activate members of the ErbB family of receptor tyrosine kinases which regulate many aspects of cell function including survival, differentiation, growth and proliferation (Falls, 2003). In the nervous system, neuregulin (NRG1) is involved in the development of neurons and glial cells (Cespedes et al., 2018), NRG2 and NRG3 have roles in synaptogenesis and synaptic function, while NRG5 and NRG6, although less extensively studied, are highly expressed in the brain where NRG6 is required for radial migration in the neocortex (Zhang et al., 2013; Mei and Nave, 2014). In contrast, the role of NRG4 in the developing brain has only recently been studied. Neocortical pyramidal neurons and striatal medium spiny neurons from mice lacking NRG4 exhibit shorter and less elaborated dendrites (Paramo et al., 2018, 2019).

Neurons acquire a polarized morphology while they migrate to cortical layers, establish connections and form functional circuits. Failure to acquire and maintain an adequate size, appropriately extend and elaborate processes and form functional synapses results in impaired neuronal function and is associated with neurodevelopmental disorders (Parenti et al., 2020). The alterations in dendritic growth and elaboration in neocortical pyramidal neurons lacking NRG4 that have previously been reported led us to explore whether these neurons display further morphologic defects caused by the loss of NRG4/ErbB4 signaling. We analyzed the cell body size of layer (L)2/3 and L5 neocortical pyramidal neurons as well as a subpopulation of interneurons (multipolar) in the motor cortex of *Nrg4*^−/−^ and *Nrg4*^+/+^ mouse brains. L5 pyramidal neurons were the most affected by the loss of NRG4, exhibiting a <20% reduction in soma size at P10. In contrast, the overall morphology of multipolar cortical interneurons was not altered, including the cell body size and dendritic length and complexity. This defect was replicated in cultured cortical pyramidal neurons, and was restored to normal by treatment with NRG4 protein in young developing neurons. These morphologic defects were associated with deficiencies in motor functions in NRG4-null mice as assessed by Rotarod performance. Overall, our results suggest that NRG4 plays an important role in the acquisition and maintenance of the appropriate morphology of a subset of neurons in the motor cortex and in motor function.

## Materials and Methods

### Animals

All animal procedures were performed in accordance with Cardiff University animal care committee’s regulation. Mice were housed in a 12/12 h light/dark cycle with access to food and water *ad libitum*. Mice lacking functional *Nrg4* expression caused by retroviral insertion of a gene trap between exons 1 and 2 were obtained from the Mutant Mouse Resource Centre, University of California Davies (CA). Mice were backcrossed from a C57BL/6 background into a CD1 background. *Nrg4*^+/+^ and *Nrg4*^−/−^ mice were generated by crossing *Nrg4*^+/−^. Male and female mice were separated after weaning (three to four weeks after birth) and kept with littermates (four to five mice per cage). For the behavioral assays, postnatal day (P)60 mice were handled for 2 d before the test. The behavioral tests were performed during the light cycle.

### Immunohistochemistry (IHC)

Wild-type P10 and P30 mice were perfused and postfixed for 16 and 3 h, respectively, in 4% paraformaldehyde in 0.12 m PBS at 4C. After fixation, brains were washed three times with PBS and sectioned using a vibratome; 50-μm sections were collected in multi-well plates, permeabilized in 0.1% Triton X-100 in PBS, heated in 10 mm sodium citrate buffer for 5 min at 95C, washed and blocked in 5% BSA, 3% donkey serum, 0.1% Triton X-100 serum in PBS (blocking solution) for 1 h at room temperature (RT) and incubated with primary antibodies in 5× diluted blocking solution as follows: anti-Nrg4 (1:100, Abcam anti-rabbit polyclonal antibody ab19247) anti-Nrg4 (20 μg/ml, Abcam anti-mouse monoclonal antibody ab239580), anti-ErbB4 (5 μg/ml, Abcam anti-mouse monoclonal antibody ab19391), anti-Fezfz (1:500, Abcam anti-rabbit polyclonal antibody ab69436), anti-Gpr88 (10 μg/ml, Novus Biologicals anti-rabbit polyclonal antibody NBP1-02330) overnight at 4C. Unbound primary antibody was washed three times with PBS, and slices were incubated with fluorophore-conjugated secondary antibodies (1:500, donkey anti-rabbit, rat, or mouse Alexa Fluor 488 and Alexa Fluor 594) for 1 h at RT. Slices were then washed three times with PBS, incubated in DAPI (1:4000) for 15 min and mounted on microscope slides using DAKO mounting medium. Sections were visualized using a Zeiss LSM 780 confocal microscope and ZEN Black software (version 2.0).

### Analysis of neuronal soma size

Golgi–Cox impregnation was performed on 150-μm coronal sections of P10, P30, and P60 *Nrg4*^+/+^ and *Nrg4*^−/−^ mouse brains by the FD Rapid GolgiStain kit (FD Neurotechnologies) according to manufacturer’s instructions. Pyramidal neuronal soma size (area and perimeter) were quantified in micrographs using FIJI (ImageJ) by tracing the outline of neuronal somata. The soma area and perimeter of a total of 90 pyramidal neurons per genotype at each age (*n* = 30 neurons per mouse, *n* = 3 mice per genotype at each age) of L2/3 and L5 of the motor cortex was measured and analyzed. Regarding interneurons, because of the heterogenicity of interneuronal morphology, three different types of interneurons were clearly identified in Golgi preparations: basket, bipolar and multipolar interneurons, the latter being the most abundant. Therefore, we quantified the soma and perimeter of multipolar interneurons in the same way as for pyramidal neurons and, in addition, we measured dendritic complexity using the Neurite tracer plug of FIJI, and the Sholl profile of reconstructed neurons, as previously described (Paramo et al., 2018). The soma area, perimeter and dendritic complexity of a total of 30 of multipolar interneurons per genotype at each age (*n* = 10 neurons per mouse, *n* = 3 mice per genotype at each age) were analyzed.

For the *in vitro* analysis of neuronal soma size, pyramidal neurons from E16 *Nrg4*^+/+^ and *Nrg4*^−/−^ cortices were cultured as previously described (Paramo et al., 2018). After 3 d *in vitro* (DIV), neurons were fluorescently labeled with calcein-AM (2 μg/ml) for 15 min at 37C. For the 9 DIV experiments, neurons were transfected after 7 DIV with a GFP plasmid (1 μg/ml) using lipofectamine for 3 h. 2 d later, neurons were fixed using 4% paraformaldehyde, washed and kept in PBS. In some experiments, CD1 or *Nrg4*^−/−^ neurons were treated with 100 or 1000 ng/ml recombinant NRG4 (Thermo Fisher Scientific). Micrographs of fluorescently labeled neurons at 3 and 9 DIV were taken using an Axiovert 200 Zeiss fluorescent microscope. The soma area of cultured pyramidal neurons was quantified using FIJI (ImageJ) as described for the Golgi images. A total of 90 neurons per age per condition from three independently generated cortical cultures were analyzed.

### Rotarod tests

Male *Nrg4*^+/+^ and *Nrg4*^−/−^ mice were tested for motor defects using the Rotarod. On the day of the experiment, mice were placed on a rod that accelerated to 40 rpm within 5 min. The latency to fall was measured across seven subsequent trials and is expressed as the time spent on the rod until the test mouse fell off, gripped to the rod, followed the rod for a full rotation or the test ended after 5 min. The performance of each individual was also compared by obtaining the slope (b) values of each curve after logarithmic regression:

(y=b log x + a).

### Elevated plus maze test

Male and female *Nrg4*^+/+^ and *Nrg4*^−/−^ mice were tested for changes in levels of anxiety using the elevated plus maze assay. On the day of the test, each mouse was placed at the center of the maze and recorded for 5 min. A tracking software (EthoVision XT, Noldus) was used to quantify the time spent in the open arms as more anxious mice spend less time there. The sum of the time spent in the left plus right open arms was calculated and compared.

### Open-field test

In addition to the elevated plus maze, anxiety was evaluated in adult male and female *Nrg4*^+/+^ and *Nrg4*^−/−^ mice using the open field test. On the day of the test, each mouse was placed at the center of an open-field arena (40 × 40 cm) in the dark and recorded for 20 min. The total distance traveled and the time spent in the center of the arena were automatically obtained by the tracking software EthoVision.

### Novel object recognition test

Changes in the ability of mice to react to novelty were evaluated using the novel object recognition test. Adult male and female *Nrg4*^+/+^ and *Nrg4*^−/−^ mice were placed in an open-field containing two equal objects attached to the floor (familiarization) and recorded for 5 min. The next day, mice were placed in the open-field containing a familiar object and a novel object and were recorded for 5 min (test). The time spent exploring either object was automatically obtained by the tracking software EthoVision. The time spent exploring either object on the familiarization day (same object) and on the test day (familiar and novel object) were plotted for comparison. To calculate the percentage of object discrimination, the time spent exploring the novel object was divided by the total time spent exploring multiple by 100.

### Statistical analysis

Data were analyzed using GraphPad Prism 8 and are expressed as mean ± SEM. For analysis of *in vivo* soma size, values were compared by one-way ANOVA with Fisher’s *post hoc* test for multiple comparisons. *In vitro* differences in soma size and rescue by recombinant NRG4 were analyzed by one-way ANOVA with Tukey’s *post hoc* test for multiple comparison. For the Rotarod test, data were compared by repeated measures ANOVA followed by Bonferroni *post hoc* test for multiple comparison. Logarithmic regression of all individual mice performance was performed to obtain slope or b values from the equation y = b log x + a; b values were then compared by an unpaired one-tailed *t* test (increase latency as increased performance). Novel object recognition data from *Nrg4*^+/+^ mice were compared after normalization by a *t* test. Adjusted *p* values were considered significant as follows: ****p < 0.0001, ***p < 0.001, **p < 0.01 and *p < 0.05.

## Results

### Nrg4 and Erbb4 mRNAs are expressed in discrete populations of cortical neurons of the anterolateral motor cortex

**Figure 1.**
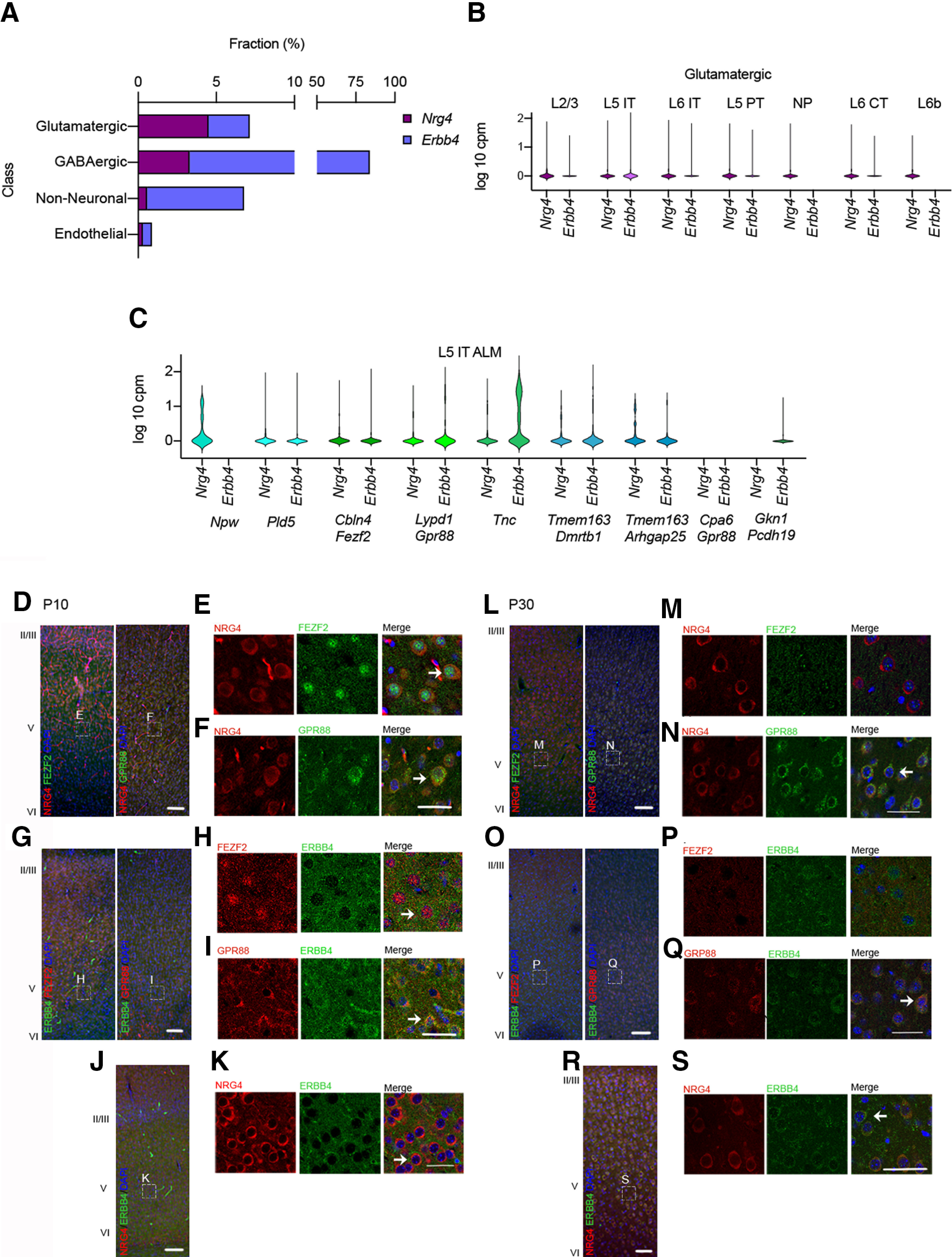
*Nrg4* and *ErbB4* mRNA expression in single cells of the adult mouse cortex obtained from RNA-sequencing public repository data. ***A***, Fraction in percentage of single cells expressing *Nrg4* (purple) and *ErbB4* (violet) in each class of cells: glutamatergic, GABAergic, non-neuronal, and endothelial. ***B***, Fraction and levels of expression of *Nrg4* and *ErbB4* mRNA in seven subclasses of glutamatergic cortical neurons. ***C***, *Nrg4* and *ErbB4* mRNA levels of expression in single cells in all the clusters in the glutamatergic neurons class from the ALM L5 IT (L5 IT ALM) subclass. P10 (***D–I***) and P30 (***L–Q***) IHC co-labeling of NRG4 and Fezf2, NRG4 and Gpr88, ErbB4 and Fezf2, and ErbB4 and Gpr88. NRG4 and ErbB4 co-expression at P10 (***J***, ***K***) and P30 (***R***, ***S***). Arrows indicate co-expression of both proteins. Dashed boxes indicate where higher magnifications were taken from. Scale bars: 100 μm (high magnification) and 50 μm (lower magnification).

NRG4 expression in different brain regions of the developing mouse brain has been recently reported (Paramo et al., 2018), while the expression of its receptor ErbB4 has long been known to be high in cortical interneurons (Mei and Nave, 2014). A shortcoming of the former study is that estimation of NRG4 mRNA levels in different brain regions does not reveal which kinds of cells express NRG4 in these regions. However, single-cell public RNA sequencing repositories, where the transcriptome of different subclasses of cells is established and the expression of particular genes can be obtained and analyzed, can be used to determine which kinds of cells express different levels of particular genes. We used the public repository published by Tasic and colleagues in 2018, where the authors analyzed cell types in the adult mouse anterior lateral motor cortex (ALM) and the primary visual cortex (VISp; Tasic et al., 2018). Tasic and colleagues established four classes of cortical cells: glutamatergic, GABAergic, non-neuronal and endothelial cells. Glutamatergic and GABAergic classes were further divided in subclasses and clusters based on the presence of specific markers or the localization of those neurons. *Nrg4* mRNA was expressed across all classes in a 4.5% fraction in glutamatergic neurons, 3.2% in GABAergic neurons, and 0.57% and 0.29% in non-neuronal and endothelial cells, respectively (Fig. 1*A*). In contrast and as previously reported, *ErbB4* mRNA was mostly expressed in GABAergic neurons (80.7%), and to a lesser extent in glutamatergic (3.2%), non-neuronal (6.2%), and endothelial (0.59%) cells. Based on the previously reported observation that the loss of NRG4 specifically affects the morphology of pyramidal neurons, we selected the “glutamatergic class” to determine the proportion of neurons in all its subclasses expressing *Nrg4* mRNA and *ErbB4* mRNA. *Nrg4* mRNA was expressed in all the glutamatergic subclasses in the following fraction: L2/3 7.2%, L4 5.7%, L5 intratelencephalic (IT) 4.08%, L6 IT 4.9%, L5 pyramidal tract (PT) 4.17%, near-projecting (NP) 1.99%, L6 corticothalamic (CT) 3.2% and L6b 2.78%, while *Erbb4* mRNA was only expressed in >1% of all the glutamatergic subclasses in L5 IT neurons (9.2%; Fig. 1*B*). Since the glutamatergic subclass with the highest fraction of neurons expressing both the ligand and the receptor mRNAs was the L5 IT subclass, we decided to further analyze the levels of *Nrg4* mRNA and *Erbb4* mRNA in each cluster from the “ALM L5 IT” subclass. We found similar levels of *Nrg4* mRNA and *ErbB4* mRNA in the majority of neurons in the following six out of the nine clusters that comprise this subclass (% *Nrg4 mRNA*; % *ErbB4* mRNA): L5 IT *Pld5* (15% and 7%), L5 IT *Cbln4 Fezf2* (17% and 7%), L5 IT *Lypd1 Gpr88* (14% and 16%), L5 IT *Tnc* (20% and 55%), L5 IT *Tmem163 Dmrtb1* (21% and 19%), L5 IT *Tmem163 Arhgap25* (31% and 9%; Fig. 1*C*).

### Nrg4 and ErbB4 are co-expressed with FEZF2 and GPR88 in L5 cortical neurons of the motor cortex during development

The above results suggest that Nrg4 and Erbb4 are at least partially co-expressed in the same type of neurons. To verify these data, we conducted immunolabeling experiments e used FEZF2, a transcription factor previously reported to be involved in dendritic arborization and the development of spines of L5 pyramidal neurons (Chen et al., 2005) and GPR88 a G-protein-coupled receptor that is expressed in the cortex to regulate multisensory integration, a function of the cortex altered in neuropsychiatric disorders (Ehrlich et al., 2018). We conducted double immune-labeling in the motor cortex of P10 and P30 wild-type mice. We observed co-expression of NRG4 with FEZF2 and GPR88 in the deep layers of the motor cortex at P10 and P30, although the levels of FEZF2 seemed to be lower at P30 (Fig. 1*D-F*,*L–N*). To further support these findings, we used CTIP2, a developmental transcription factor, and the major downstream FEZF2 effector (Chen et al., 2008). In the motor cortex of wild-type mice at P10, we observed co-expression of FEZF2 and CTIP2, as expected, but more importantly of CTIP2 and NRG4 and CTIP2 and ERBB4, although few cells with no co-expression were also observed (Fig. 7). Furthermore, we also observed co-localization of ERBB4 and the two markers tested above, FEZF2 and GPR88 at P10 and P30 (Fig. 1*G–I*,*O–Q*). Lastly, we also observed co-expression of NRG4 and ERBB4 in the motor cortex at P10 and P30 (Fig. 1*J*,*K*,*R*,*S*). Taken together, these observations corroborate the single-cell RNA sequencing data, and show that Nrg4 contributes to pyramidal neuronal development. These results from our IHC experiments strengthen and extend the conclusion about the contribution of NRG4 to developing pyramidal neurons that is likely to be mediated either partly or entirely by an autocrine mechanism, although from the lack of co-expression in a proportion of neurons we cannot rule out the participation of a paracrine mechanism.

### Absence of NRG4 causes a decrease in soma size of pyramidal neocortical neurons *in vivo*

**Figure 2.**
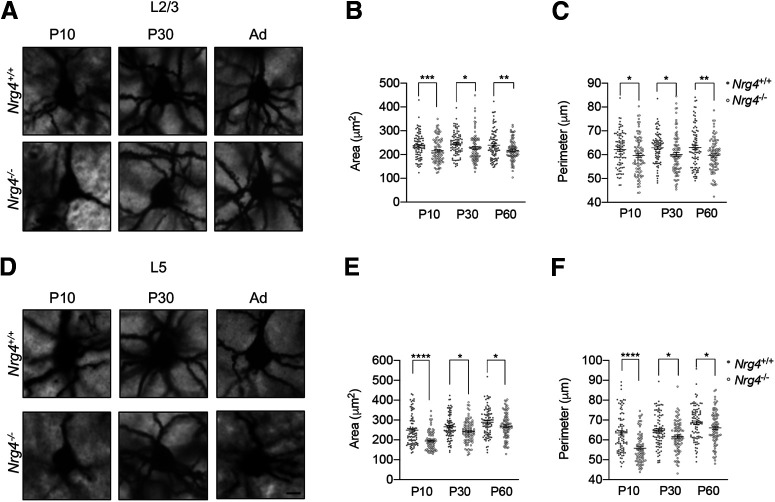
Neuronal soma size is decreased in motor cortex L2/3 and L5 neocortical pyramidal neurons of *Nrg4*^−/−^ mice. Representative micrographs of Golgi-impregnated pyramidal neurons in L2/3 (***A***) and L5 (***D***) of the motor cortex at P10, P30, and P60 *Nrg4*^+/+^ and *Nrg4*^−/−^ mice. Scale bar: 25 μm. ***B***, ***E***, Quantification of the area and perimeter. Data are mean ± SEM. ***C***, ***F***, One-way ANOVA with Fisher’s *post hoc* test for multiple comparison, ****p < 0.0001, ***p < 0.001, **p < 0.01, *p < 0.05.

It has been previously reported that the loss of NRG4 impairs the development of dendrites, reducing the size and complexity of the dendritic tree of pyramidal neurons of the motor cortex (Paramo et al., 2018). To further investigate the morphologic effects caused by the loss of NRG4 expression in neocortical pyramidal neurons, we examined cell size of pyramidal neurons in L2/3 and L5 in *Nrg4*^−/−^ and *Nrg4*^+/+^ mice at two postnatal ages (P10 and P30) and in adults (P60). Golgi preparations of coronal sections from the most rostral part of the neocortex, including the frontal/motor cortex and the most rostral region of the somatosensory cortex were used to measure soma size (area and perimeter) of pyramidal neurons. In L2/3, we found similar significant reductions in both soma size and perimeter in *Nrg4*^−/−^ mice compared with *Nrg4*^+/+^ littermates at P10 (area *p *=* *0.0105; perimeter *p *=* *0.0305), P30 (area *p *=* *0.0267; perimeter *p *=* *0.0123), and P60 (area *p *=* *0.0079; perimeter *p* = 0.0040; Fig. 2*A–C*). The average reductions in pyramidal neuron soma area in *Nrg4*^−/−^ mice were 8.5%, 8.8%, and 4% in P10, P30, and P60 mice, respectively. In L5, we also observed significant reductions in pyramidal neuron soma area and perimeter in *Nrg4*^−/−^ mice compared with *Nrg4*^+/+^ littermates at all stages studied: P10 (area *p *<* *0.0001; perimeter *p *<* *0.0001), P30 (area *p *=* *0.0115; perimeter *p *=* *0.0123), and P60 (area *p *=* *0.0344; perimeter *p *=* *0.0404; Fig. 2*D–F*). However, the reductions at P10 were quantitatively greater and more highly significant than at later ages. At P10, there was a 22% reduction in the soma area of L5 pyramidal neurons in *Nrg4*^−/−^ mice compared with *Nrg4*^+/+^ littermates.

### Absence of NRG4 does not significantly affect soma size of neocortical multipolar interneurons *in vivo*

**Figure 3.**
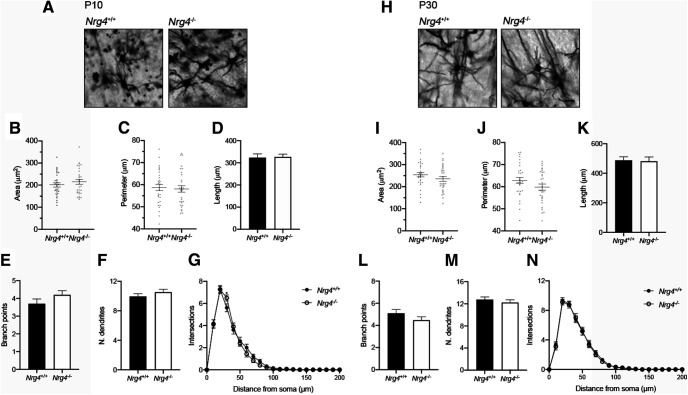
Unaffected neuronal morphology and soma size of multipolar interneurons from *Nrg4*^−/−^ mice. ***A***, ***H***, Representative micrographs of Golgi-stained multipolar interneurons from *Nrg4*^+/+^ and *Nrg4*^−/−^ mice at P10 and P30, respectively. Scale bar: 25 μm. Total area (***B***, ***I***), perimeter (***C***, ***J***), total dendritic length (***D***, ***K***), number of branch points (***E***, ***L***), number of dendrites (***F***, ***M***), and Sholl analysis (***G***, ***N***). Data are mean ± SEM.

To determine whether the effect of NRG4 deficiency on soma size was pyramidal neuron specific or whether it affected other neuronal types in the neocortex, we evaluated the soma size of a population of interneurons. Bipolar, basket, and multipolar interneurons (Ascoli et al., 2008) were evident in our Golgi preparations, but because multipolar interneurons were the most abundant we focused our analysis on these neurons. In addition to assessing soma size (area and perimeter), we also quantified the size and complexity of their dendritic arbors (total dendrite length, total number of branch points and Sholl profiles). We did not find any significant differences in cell body size (Fig. 3*A–C*,*H–J*). The area and perimeter of *Nrg4*^+/+^ interneurons were (mean ± SEM) 201.2 ± 9.6 and 58.73 ± 1.4 μm^2^, respectively, while *Nrg4*^−/−^ area and perimeter were (mean ± SEM) 214.8 ± 11.13 and 58.08 ± 1.48 μm^2^ (Fig. 3*B*,*C*). At P10 the total dendritic length was (mean ± SEM) 323.2 ± 17 and 326.6 ± 12.46 μm^2^ for *Nrg4*^+/+^ and *Nrg4*^−/−^, respectively (Fig. 3*D*), while the number of branching points was (mean ± SEM) 3.7 ± 0.26 for *Nrg4*^+/+^ and 4.2 ± 0.24 for *Nrg4*^−/−^ (Fig. 3*E*). The total number of dendrites was also similar (mean ± SEM) 9.9 ± 0.35 in *Nrg4*^+/+^ multipolar interneurons and 10.5 ± 0.38 in *Nrg4*^−/−^ (Fig. 3*F*). The lack of differences was reflected in the almost completely overlapping Sholl profiles (Fig. 3*G*). At P30, A small but non-significant (*p *=* *0.1988) difference was observed in the total soma area between genotypes (mean ± SEM: 254.6 ± 10.25 vs 235.4 ± 10.63 μm^2^; Fig. 3*I*). The dendritic outgrowth was similar among genotypes (Fig. 3*K–N*). Taken together, the above findings suggest that NRG4 plays an important role in promoting and maintaining soma size of pyramidal neurons in the developing neocortex, especially those of L5, without significantly affecting the size and dendritic morphology of multipolar interneurons.

### Neocortical pyramidal neurons cultured from *Nrg4*^−/−^ mice replicate the small soma phenotype

**Figure 4.**
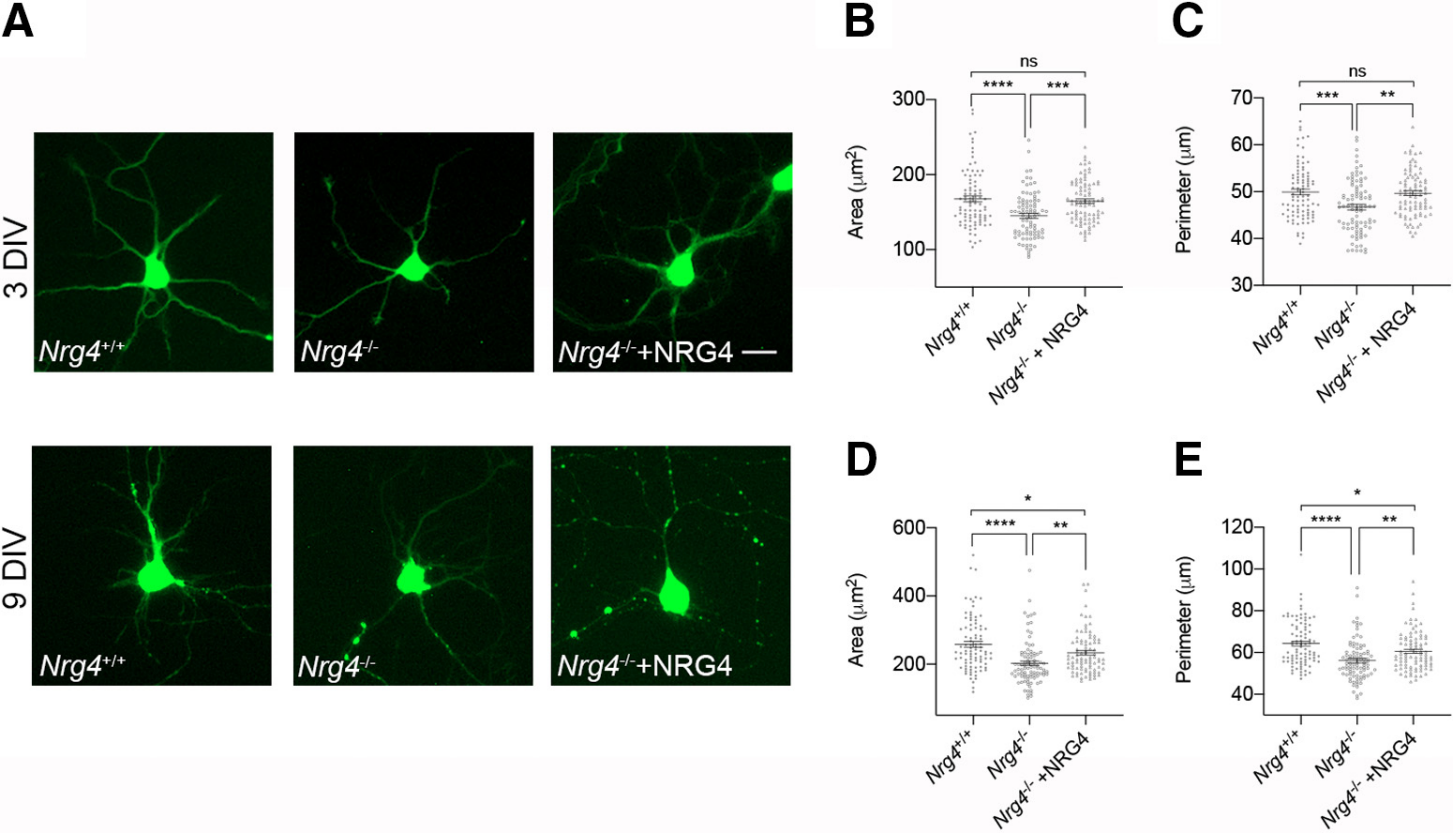
Neuronal soma size is reduced *in vitro* in *Nrg4*^−/−^ pyramidal neurons and it is rescued by NRG4 treatment. ***A***, Representative micrographs of cortical pyramidal neurons from *Nrg4*^+/+^ and *Nrg4*^−/−^ embryos at 3 and 9 DIV stained with calcein-AM (upper panel) and transfected with GFP (lower panel), respectively. *Nrg4*^−/−^ neurons were treated with recombinant NRG4 (100 ng/ml). Scale bar: 20 μm. Quantification of the area (***B***, ***D***) and perimeter (***C***, ***E***) of 90 neurons per condition. Data are mean ± SEM. One-way ANOVA with Tukey’s *post hoc* test for multiple comparison, ****p < 0.0001, ***p < 0.001, **p < 0.01, *p < 0.05, ns, not significant.

To determine whether the small soma phenotype observed *in vivo* in *Nrg4*^−/−^ pyramidal neocortical neurons is exhibited by pyramidal neurons cultured from *Nrg4*^−/−^ mice and to ascertain whether this defect can be rescued by recombinant NRG4 treatment, we established cortcal cultures from E16 *Nrg4*^+/+^ and *Nrg4*^−/−^ embryos. Pyramidal soma size (area and perimeter) was assessed after 3 and 9 d. This analysis was facilitated by labeling the cells with either calcein-AM after 3 d in culture or by GFP transfection after 7 d in culture for analysis at 9 DIV. After 3 and 9 DIV, soma area and perimeter were significantly smaller in cultures established from *Nrg4*^−/−^ embryos compared with those established from *Nrg4*^+/+^ embryos (Fig. 4). At 3 DIV, a 13.3% reduction in soma area in *Nrg4*^−/−^ (*p *<* *0.0001) and a 6.3% reduction in perimeter (*p *=* *0.0003) of pyramidal neurons was observed and it was significantly rescued by recombinant NRG4 (100 ng/ml; area *p *=* *0.0002; perimeter *p *=* *0.0011; Fig. 4*A–C*). No significant differences were observed in the soma area (mean ± SEM: 167.6 ± 4.04 vs 164.6 ± 2.94 μm^2^, respectively; adjusted *p* = 0.8106) or in the perimeter (mean ± SEM: 49.93 ± 0.6 vs 49.65 ± 0.51 μm^2^, respectively, adjusted *p* = 0.9343) between *Nrg4*^+/+^ and *Nrg4*^−/−^+NRG4 neurons, which indicates full rescue by NRG4.

At 9 DIV, the soma of *Nrg4*^−/−^ pyramidal neurons was 21.6% smaller (area *p *<* *0.0001; perimeter *p *<* *0.0001), and significantly rescue by NRG4 (area *p *=* *0.0090; perimeter *p *=* *0.0069). However, NRG4 did not fully rescue the reduction in soma area and perimeter in this case, since *Nrg4*^+/+^ versus *Nrg4*^−/−^+NRG4 values were still significantly different (mean area ± SEM: 258.3 ± 8.3 vs 233.1 ± 6.49 μm^2^, respectively; adjusted *p* = 0.0384; mean perimeter ± SEM: 64.25 ± 1.31 vs 60.62 ± 0.98 μm^2^, respectively; adjusted *p* = 0.0378).

These results show that the small soma pyramidal neuron phenotype observed *in vivo* in NRG4-deficient mice is replicated in cultured neurons and is fully rescued in young developing neurons and partially rescued in more mature neurons by soluble NRG4.

### Defects in motor performance of mice lacking *Nrg4*

**Figure 5.**
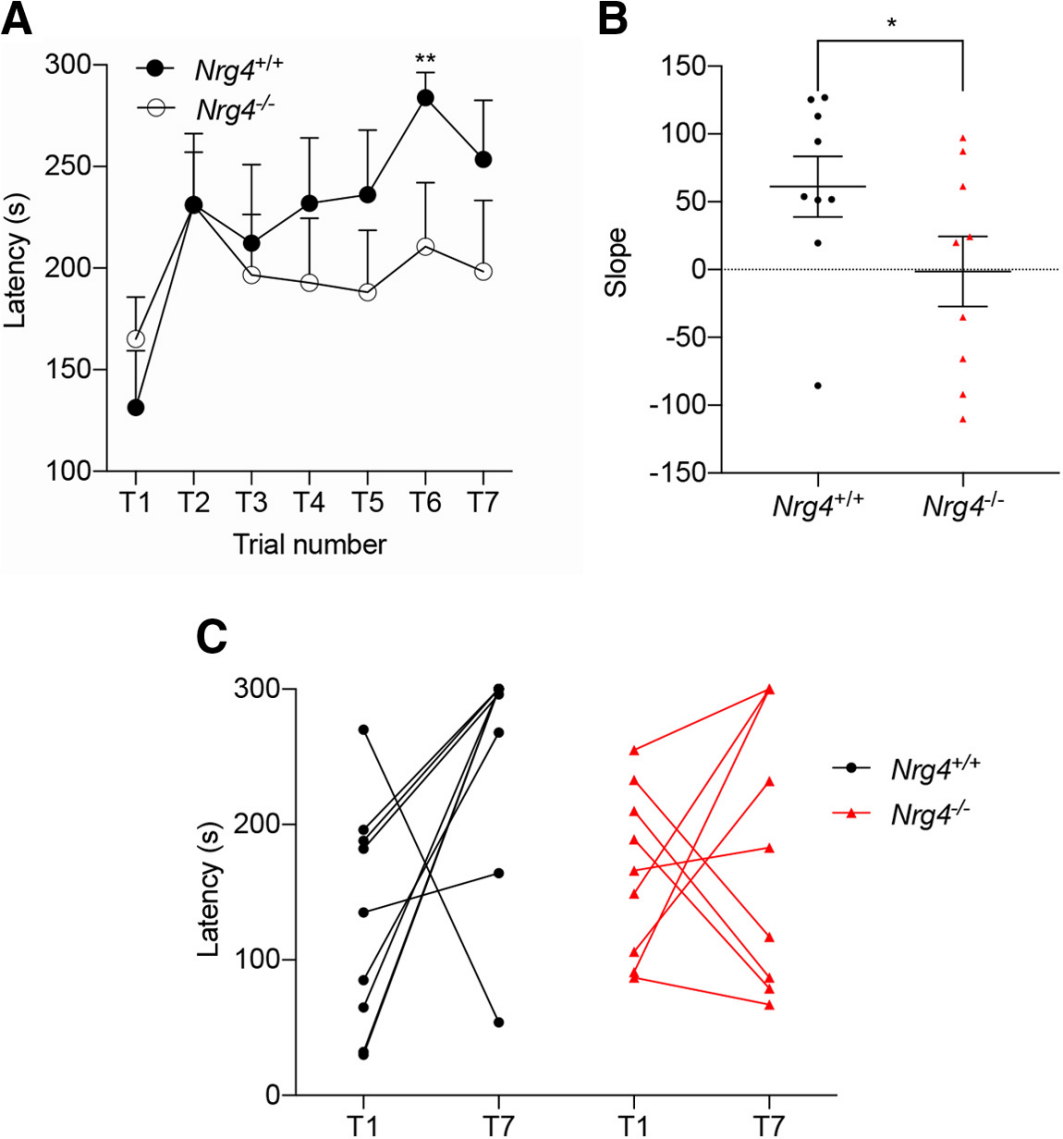
The loss of NRG4 impairs motor skills. ***A***, *Nrg4*^+/+^ P60 male mice spent significantly more time on the accelerating Rotarod after trial 6 compared with *Nrg4*^−/−^ mice. Data are mean ± SEM. Repeated measurements ANOVA with Bonferroni’s multiple comparison test versus T1; **p < 0.01 ***B***, Comparison of slope values from each animal tested obtained after logarithmic regression of each individual performance. Horizontal lines represent mean ± SEM. Unpaired *t* test *p < 0.05. ***C***, Traces of individual performance (T1–T7).

The morphologic defects observed in motor cortex pyramidal neurons led us to evaluate whether these alterations have an impact on the ability of mice to execute a motor task, such as the Rotarod test. We evaluated P60 *Nrg4*^+/+^ and *Nrg4*^−/−^ mice performance, measured as the time spent on the rod over time (seven trials, T1–T7). *Nrg4*^+/+^ performance improved with subsequent trials and the latency at T6 was significantly different when compared with T1 [latency (mean ± SEM) at T1 = 131.4 ± 27.9 s vs latency at T6 = 283.7 ± 12.4 s, *p *=* *0.0078; Fig. 5*A*]. However, *Nrg4*^−/−^ mice did not improve their performance (latency (mean ± SEM) at T1 = 167.5 ± 21.1 vs 220.5 ± 29.5 s at T6 and 209.5 ± 32.9 s at T7; Fig. 3*A*). The average slope values from each individual animal tested acquired after logarithmic regression from *Nrg4*^+/+^ and *Nrg4*^−/−^ were significantly different (*p *=* *0.0425; Fig. 5*B*). Individual traces of T1 and T7 from all the animals tested are shown in Figure 3*C*. These results suggest that the morphologic defects in pyramidal neocortical motor cortex neurons caused by the loss of *NRG4* impact the motor ability of mice.

### No differences in anxiety levels or response to novelty

**Figure 6.**
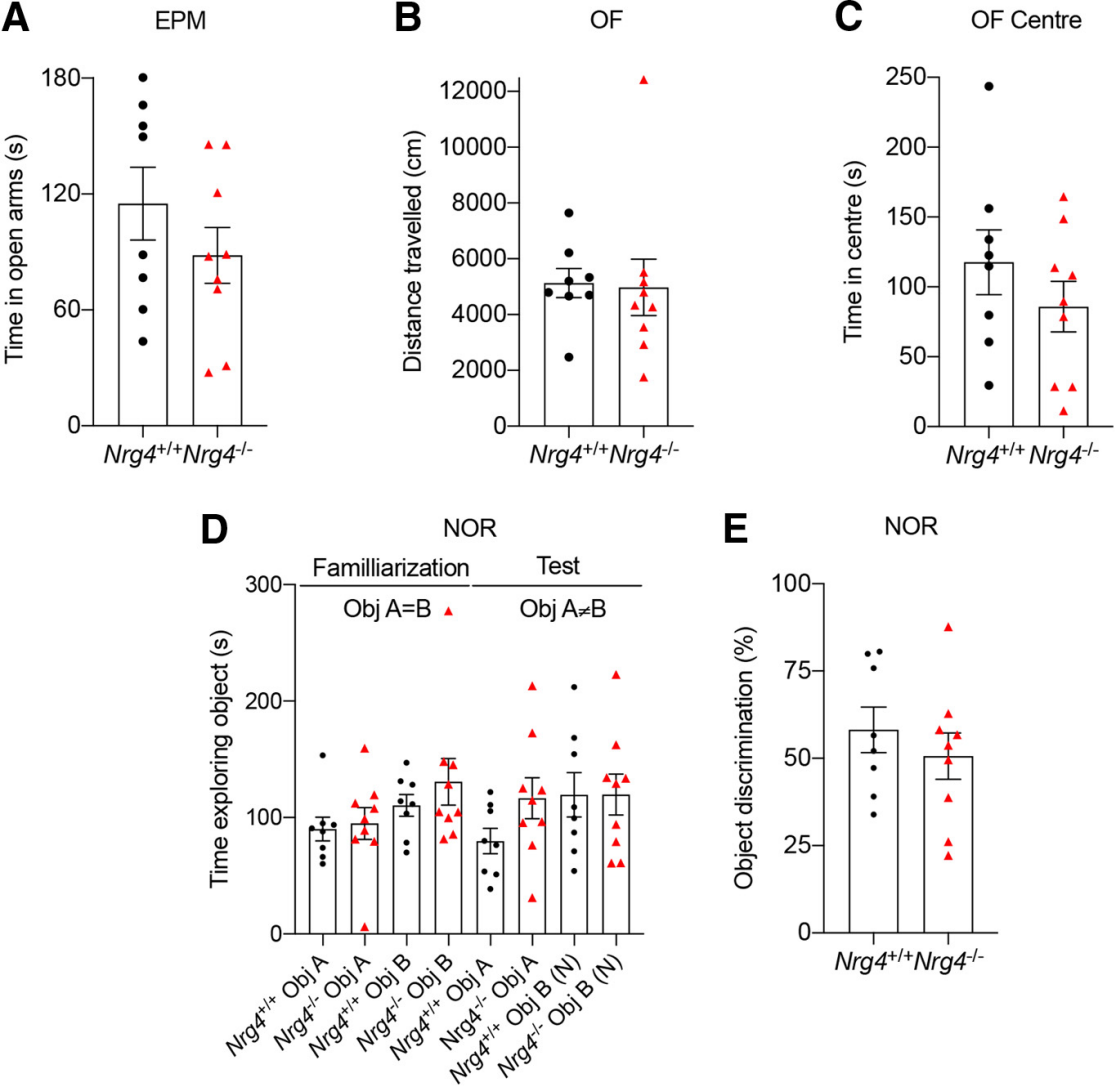
The loss of NRG4 does not affect anxiety or response to novelty. ***A***, Time spent in the open arms of the elevated plus maze (EPM) was no different among P60 *Nrg4*^+/+^ and *Nrg4*^−/−^ mice. ***B***, Total distance traveled and time spent in the center (***C***) of the open-field was similar between *Nrg4*^+/+^ and *Nrg4*^−/−^ mice. Novel object recognition (NOR) test. ***D***, P60 *Nrg4*^−/−^ mice spent a similar amount of time exploring a familiar object and a novel object. Data are mean ± SEM. ***E***, The object discrimination percentage was similar between genotypes (calculated as the total amount of time spent exploring the novel object divided by the total time spent exploring multiple by 100).

The defects observed in the morphology of pyramidal neurons of the motor cortex caused a defect in motor skills in adult mice. We also evaluated whether the lack of NRG4 affected other behavior. We evaluated anxiety levels using the elevated plus maze and open-field test as well as the response to novelty using the novel object recognition test between genotypes. Adult (P60) *Nrg4*^−/−^ mice spent slightly less time in the open arms of the maze but this decrease was not significant (Fig. 6*A*). No differences were observed in the distance traveled (Fig. 6*B*) or in the time spent in the center of the open-field (Fig. 6*C*). When exposed to a novel object, the *Nrg4*-null mice spent a similar amount of time (mean ± SEM) exploring the familiar (116.6 ± 17.61 s) and novel object (119.7 ± 17.53 s), while *Nrg4*^+/+^ mice spent more time exploring the novel (119.4 ± 19.06 s) than the familiar object (79.77 ± 10.86 s), but this difference was not significantly different (Fig. 6*D*). However, this increase was significantly different (*p *=* *0.0462) when the data from *Nrg4*^+/+^ mice were normalized to the time spent exploring the familiar object in the familiarization day, and then compared by an unpaired *t* test (mean ± SEM) 0.95 ± 0.14 for the familiar object on test day versus 1.96 ± 0.54 for the novel object on the test day. These observations were reflected in the object discrimination percentage, where the percentage in *Nrg4*^+/+^ versus *Nrg4*^−/−^ mice was slightly reduced from 58.15 ± 6.5% to 50.6 ± 6.6%, but no significantly different (*p *=* *0.4366; Fig. 6*E*).

**Figure 7.**
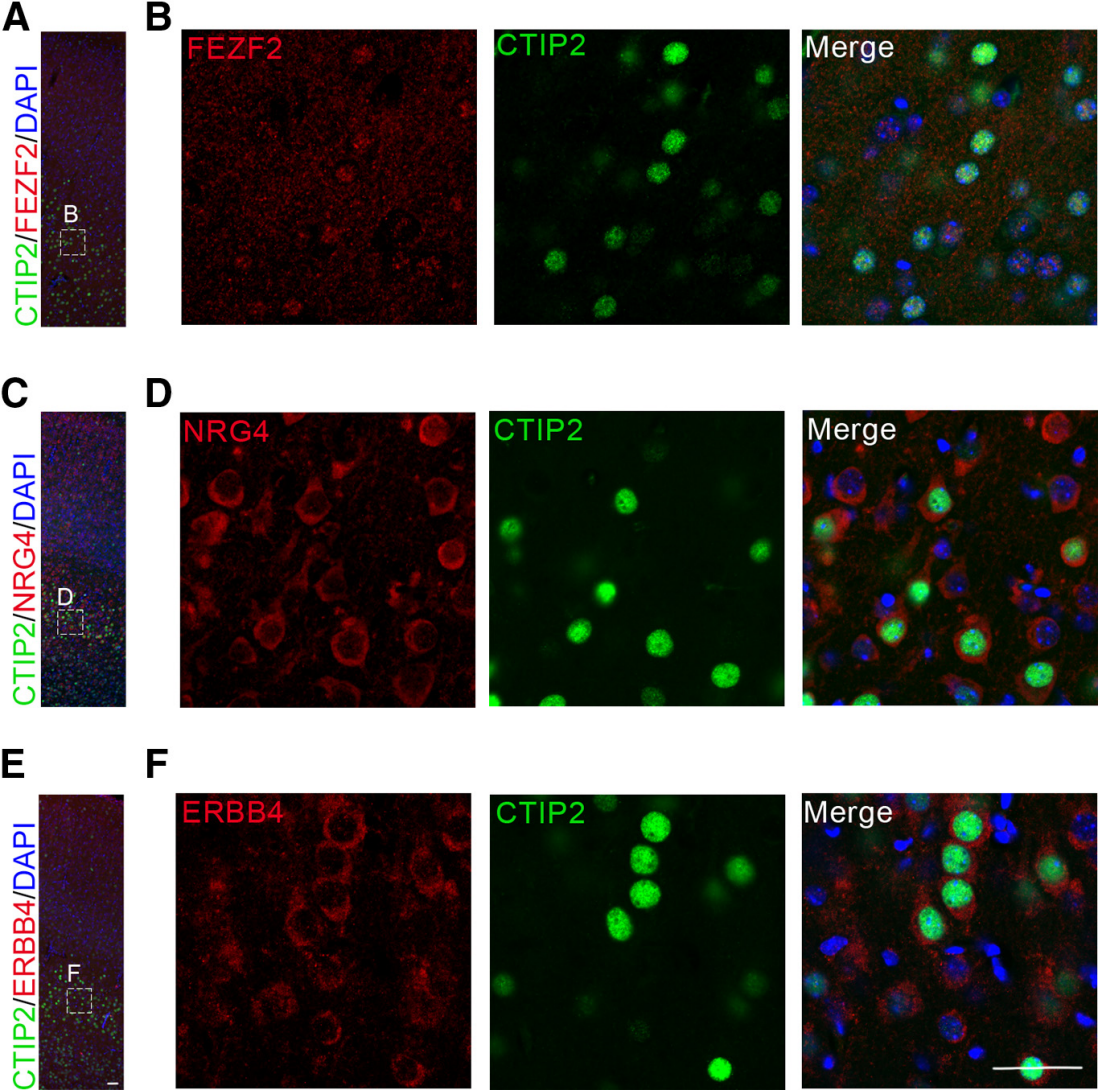
NRG4 and ErbB4 co-expression with Ctip2, a major FEZF2 effector, in the motor cortex of young postnatal brain. CTIP2 and FEZF2 (***A***, ***B***), CTIP2 and NRG4 (***C***, ***D***), and CTIP2 and ERBB4 (***E***, ***F***) co-labeling in neurons from the motor cortex at P10. Scale bar: 50 μm.

## Discussion

Neuregulins play a diverse and critical role in the development of the nervous system. NRG4 function in the developing motor cortex contributes to the dendritic outgrowth and complexity of pyramidal neurons. However, further morphologic defects in pyramidal neurons or other type of cortical neurons lacking NRG4 have not been investigated and the functional consequences of such defects have not been studied. Neuronal morphologic defects greatly impact the function of the circuits, causing neurodevelopment disorders. Thus, defining the morphologic defects caused by altered NRG4/ErbB4 signaling and their functional consequences aids in our understanding of the crucial mechanisms required for normal cortical development and function in the adulthood. In addition, the identification of more specifically affected populations of neurons allows the development of targeted interventions to prevent these defects. In this study, we analyzed the cell soma of neocortical pyramidal and multipolar interneurons of *Nrg4*^+/+^ and *Nrg4*^−/−^ mice motor cortex at different stages to identify whether the loss of NRG4 affects soma size, and whether this effect was general or specific to certain populations of neurons. We found an important decrease in the soma size of L2/3 and more significantly of L5 pyramidal neurons with no changes in the size of multipolar interneurons or L6 pyramidal neurons (not shown) lacking NRG4. Data obtained from a single-cell RNA-sequencing public repository, show that *Nrg4* and *ErbB4* mRNAs were mostly co-expressed in glutamatergic L5 neurons of the adult ALM suggesting that a cell-autonomous NRG4/ErbB4 function is required to maintain the soma size of L5 pyramidal neurons. Furthermore, and to validate these observations, double inmuno-labeling of pyramidal neurons from the motor cortex showed that NRG4 and ErbB4 are co-expressed with markers that define specific subpopulations of excitatory neurons of the anterolateral motor cortex, such as Fezf2 and Grp88. At P10, Fezf2 and Gpr88 were found to be co-expressed with both ErbB4 and NRG4. To further strengthen these observations, we also found co-expression with the Fezf2-downstream effector Ctip2 for both NRG4 and ErbB4, although neurons with no co-expression of both markers were also found. At P30, the number of Fezf2-positive neurons decreased, but Gpr88 was still found to be co-expressed with ErbB4 and NRG4. In addition, ErbB4 and NRG4 were also found to be co-expressed in L5 motor cortex pyramidal neurons. The observation that only a proportion of neurons co-expressed both markers and the ligand or receptor suggests that a paracrine mechanism cannot be ruled out.

The mechanisms controlling cell body size in postmitotic neurons function differently than in dividing cells since in the later these pathways also regulate cell cycle progression and division. However, the observation that this effect is also observed *in vitro* suggests that NRG4 may be acting in a cell-autonomous manner to regulate neuronal soma size by modulating an intrinsic mechanism. To date, none of the neuregulins nor ErbB4 itself has been reported to control neuronal soma size in the CNS. NRG1 cystein-rich domain isoform, the most abundant isoform in the brain, is required to maintain an excitatory/inhibitory balance between interneurons and pyramidal cells (Agarwal et al., 2014), whereas NRG2, expressed at P7 in the hippocampus and neocortex, localizes to the soma and dendrites of hippocampal neurons (Longart et al., 2004), where it downregulates glutamatergic transmission by promoting the internalization of GluN2B-containing NMDA receptors (Vullhorst et al., 2015). Deletion of *ErbB4* in neocortical excitatory neurons alters dendritic spine maturation (Cooper and Koleske, 2014). However, these studies did not look at the effects of neuregulin deletion or overexpression on overall neuronal morphology nor in the potential role of neuregulins in the acquisition and maintenance of neuronal size. In mouse models of genetic disorders that result in dendritic abnormalities such as Rett syndrome, a decrease in the soma size of hippocampal neurons was observed (Rangasamy et al., 2016), while loss of function mutations in the tuberous sclerosis complex (TSC), a negative regulator of the mTOR/Akt pathway, increase the soma size of hippocampal pyramidal neurons but decrease the density of dendritic spines causing alterations in glutamatergic transmission (Tavazoie et al., 2005). Likewise, depleted c-Jun N-terminal kinase 1 signal increases L5 motor cortex pyramidal neuron dendritic length and increases soma size (Komulainen et al., 2014). The decrease in dendritic architecture observed in *Nrg4* knock-out pyramidal neocortical neurons is consistent with a decrease in soma size, since there is a correlation between dendritic and cell body size (van Pelt et al., 1996). Furthermore, the lack of differences between soma size and dendritic complexity in a subpopulation of interneurons in *Nrg4* knock-out mice supports this observation. The possibility of alterations in the morphology of other types of interneurons remains, since multipolar interneurons are only a fraction of this diverse class of neurons. In our analysis, the multipolar interneurons we measured are biochemically defined by calbindin expression. This population of interneurons is also transcriptomically defined by parvalbumin and somatostatin expression, according to the clusters described in the single-cell RNA sequencing study. All the interneurons in this cluster express *ErbB4* and in fact, *NRG3* is expressed in 56%, *NRG2* in 17%, while *NRG1* and *NRG4* are expressed only in 4.5% and 3.5% of these interneurons, respectively. The levels of NRG3 and NRG2 in these interneurons with high ErbB4 expression may explain why the signaling can be maintained in these neurons and thus why the size of the soma is not affected. The more significant decrease in soma size we observed, that is, in L5 pyramidal neurons, correlated with the levels of *Nrg4* and *ErbB4* mRNA co-expression. L5 IT pyramidal neurons project axons within the telencephalon. In contrast to cortical sensory areas, L5 pyramidal neurons in the anterolateral motor cortex display slower dynamics and are involved in controlling movement planning, among other functions (Svoboda and Li, 2018). Consistent with the phenotypical defects observed in *Nrg4* knock-out neurons, the clusters with similar levels of receptor and ligand co-expression are defined by markers related to cytoskeletal dynamics, synaptic function, dendritic arborization and spine formation, migration and axonal development, and have been reported to be altered in neurodevelopmental disorders. For example, *Fezf2* is a transcription repressor that is implicated in the development of dendritic arborization and spines of large L5 pyramidal neurons (Chen et al., 2005) a population of neurons affected in schizophrenia (Shepherd, 2013). Interestingly, *ErbB4* has been reported to be a susceptibility gene for this disease (Silberberg et al., 2006). In addition, *Gpr88* encodes a G-protein-coupled receptor that is expressed in the cortex to regulate multisensory integration, a function of the cortex altered in neuropsychiatric disorders (Ehrlich et al., 2018). Lastly, *Arhgap25* encodes a Rho GTPase activating protein (GAP), that acts as a negative regulator of Rho GTPases, proteins implicated in actin remodeling, cell polarity, and migration (Hodge and Ridley, 2016). GAP activity is required to regulate Rac1 activation since alterations in this pathway have been implicated in intellectual disability (Lelieveld et al., 2016), a neurodevelopmental disorder characterized by defects in network connectivity and unbalanced excitation and inhibition in the cerebral cortex.

Importantly, the morphologic defects caused by the loss of NRG4 caused an impairment in the motor skills of adult mice, while total motor activity, anxiety and response to novelty were unaffected. This can be in part because of the fact that the brain areas mediating these behaviors are unaffected in *Nrg4* knock-out mice, or maybe other neuregulins can compensate for the loss of NRG4 in specific areas where these neuregulins are more abundant. In agreement with our observation, genetic models where the function of downstream effectors of ErbB4 signaling such as Akt and mTOR is inhibited in a brain-specific manner or by rapamycin treatment display impair Rotarod performance (Thomanetz et al., 2013; Bergeron et al., 2014). Importantly, together with the requirement of NRG4 for maintaining soma size, the defects observed in overall dendritic outgrowth are likely to be a major contributor of the defects in performing the Rotarod task. Although we cannot completely rule out a contribution from the cerebellum in the motor defects observed in the *Nrg4*-null mice, we have found that at P12, Purkinje neurons from *Nrg4* knock-out mouse brains did not display any significant morphologic defects (data not shown). However, morphologic or any other physiological alterations in other cell types that comprise the cerebellum cannot be discarded as contributors to the motor phenotype. We therefore cannot attribute the motor deficiencies to the morphologic defects observed in the motor cortex without considering that alterations in the cerebellum can also be accounted for these deficiencies in performing a motor task.

Further studies where NRG4 is specifically deleted in pyramidal neurons will inform more as to the requirement of NRG4/ErbB4 signaling for circuit formation and function. Likewise, the generation of transgenic lines were *Nrg4* is depleted specifically in other populations of CNS neurons may be informative.

Taken together, our observations indicate that NRG4 plays a broader biological role in the control of neuronal morphology. Our results show that NRG4 is important for the development and maintenance of the morphology of neocortical pyramidal neurons of the motor cortex, and that some populations rely more on its expression early during development to acquire normal size. Besides its role in development, its expression in motor areas in the adult mouse brain in transcriptomically defined populations of pyramidal neurons shows that it may be required for the specific adequate function of these neurons and perhaps its loss or altered expression, which in turns would affect NRG4/ErbB4 signaling, may contribute to the pathogenesis of neurodevelopmental or psychiatric disorders.

## Synthesis

Reviewing Editor: Eran Perlson, Tel Aviv University Decisions are customarily a result of the Reviewing Editor and the peer reviewers coming together and discussing their recommendations until a consensus is reached. When revisions are invited, a fact-based synthesis statement explaining their decision and outlining what is needed to prepare a revision will be listed below. The following reviewer(s) agreed to reveal their identity: Oded Behar.

In this study, the authors investigated the role of neuregulin-4 (NRG4) in the development of cortical neuron soma size. Using neureguilin-4 knockout (Nrg4-/-) animals in combination with Golgi staining, it was demonstrated that layers 2/3 and 5 pyramidal neurons displayed significantly reduced soma area and perimeter at different early postnatal stages. Layer 5 pyramidal neurons from the mutant mice in the motor cortex showed a more severe soma size difference at P10 compared to WT littermates. Primary cortical cultured neurons from E16.5 Nrg4-/- embryos also displayed reduced soma size compared to WT neurons and, the phenotype was at least partially rescued when exogenous soluble NRG4 were applied. The authors analyzed the single cell RNA-sequencing data from both glutamatergic and GABAergic neurons in the adult mouse anterior lateral motor cortex from a previously published study, and showed that both Nrg4 and its receptor ErbB4 are expressed in both types of neurons, proposing an autocrine model of action. Finally, using the rotarod test the authors showed that the Nrg4-/- exhibit motor learning deficits compared to WT control animals.

Overall the demonstration of motor defects and reduced soma size of pyramidal neurons during development is new and interesting. However, there are several issues that the authors should address before publications:

1. The use of the single cell data from adult motor neurons is a poor rationale and a weak connection to this current developmental study. The single cell RNA-Seq data can still be useful as it showed that different L5 IT ALM subgroups expressed other genes (Fezf2, Tnc, Gpr88, Arhgap25), when deleted the animals display dendritic arborization phenotypes similar to the Nrg4-/- mice, or have been implicated in neurological disorders related to cortical development. At the very minimum, the authors should use anti-NRG4 or anti-ErbB4 antibodies to double immuno-label with anti-Fezf2 or anti-Gpr88, etc. (as all these antibodies are commercially available) in P0, P10 and P30 pyramidal neurons in the motor cortex. This will better link the role of Nrg4-ErbB4 signaling in cortical motor neuron development and strength the current study.

2. The rotarod phenotype seemed very specific to the Nrg4-/- animals as other behavioral tests (elevated plus maze, open field and novel object recognition) showed that the mutants have normal anxiety levels and memory. However, from a previous study by Paramo et al. (2018), it was shown that both Nrg4 and ErbB4 mRNAs are also expressed in the cerebellum at P0. Nrg4 mRNA levels in the cerebellum are even higher than the cortex. Therefore, the motor learning deficits exhibited by the Nrg4-/- animals could be due to a contribution from the cerebellum. There is a conditional Nrg4 knockout mouse line available (Nugroho et al., 2018). It is noted that during this time there might be difficulty in obtaining mice from other labs or even repository. However, the authors should acknowledge this flaw in their analysis and modify the interpretation of their results to include the cerebellum as a likely contribution to the rotarod phenotype.

3. The possible linkage between the reduced soma size and the motor defects is not clear and likely to be the result of reduced dendritic tree reported in previous paper.

4. The suggested autocrine model in which both the receptors and ligands are expressed by the same neurons is not well substantiated. This claim is based on a bioinformatics analysis of single cell RNA seq. In this analysis they find that about 75% of GABAergic neurons, mostly in L5, express Erbb4 while some of these cells also express Nrg4. This clearly does not prove their model. There was no attempt to support this model either by double staining of receptor and ligand in sections or even in vitro by using a blocking antibody (or other means of inhibition) to block Nrg4 in WT and test soma size.

Finally, here are a couple of minor concerns.

The quantifications in Fig. 3B-E shows statistical significance only between WT versus Nrg4-/- and Nrg4-/- versus Nrg4-/- + NRG4, was there statistical significance between WT versus Nrg4-/- + NRG4? If there is a full rescue of the soma size with the addition of exogenous NRG4, then no significance is expected, but if it is only a partial rescue then there could still be a significance between the two conditions. Statistical analysis between WT versus Nrg4-/- +NRG4 is needed.

The single cell data shown in Fig. 4 should be shown first, along with new double immuno-labeling results of Nrg4/ErbB4 with Fezf2 or Gpr88, etc. at early postnatal ages.

## Author Response

Dear Dr. Perlson,

Many hanks for your letter and the reviewer’s comments regarding our manuscript "Neuregulin-4 is required for maintaining soma size of pyramidal neurons in the motor cortex,"(eNeuro-NWR-0288-20). Our response to these comments and the changes we have made to the manuscript, including experimental data, are outlined point-by-point below. I trust you will find our revised manuscript suitable for publishing in eNeuro.

Best regards,

Corresponding author on behalf of the authors

Synthesis of Reviews:

Computational Neuroscience Model Code Accessibility Comments for Author (Required):

n/a

Significance Statement Comments for Author (Required):

n/a

Comments on the Visual Abstract for Author (Required):

n/a

Synthesis Statement for Author (Required):

In this study, the authors investigated the role of neuregulin-4 (NRG4) in the development of cortical neuron soma size. Using neureguilin-4 knockout (Nrg4-/-) animals in combination with Golgi staining, it was demonstrated that layers 2/3 and 5 pyramidal neurons displayed significantly reduced soma area and perimeter at different early postnatal stages. Layer 5 pyramidal neurons from the mutant mice in the motor cortex showed a more severe soma size difference at P10 compared to WT littermates. Primary cortical cultured neurons from E16.5 Nrg4-/- embryos also displayed reduced soma size compared to WT neurons and, the phenotype was at least partially rescued when exogenous soluble NRG4 were applied. The authors analyzed the single cell RNA-sequencing data from both glutamatergic and GABAergic neurons in the adult mouse anterior lateral motor cortex from a previously published study, and showed that both Nrg4 and its receptor ErbB4 are expressed in both types of neurons, proposing an autocrine model of action. Finally, using the rotarod test the authors showed that the Nrg4-/- exhibit motor learning deficits compared to WT control animals.

Overall the demonstration of motor defects and reduced soma size of pyramidal neurons during development is new and interesting. However, there are several issues that the authors should address before publications:

1. The use of the single cell data from adult motor neurons is a poor rationale and a weak connection to this current developmental study. The single cell RNA-Seq data can still be useful as it showed that different L5 IT ALM subgroups expressed other genes (Fezf2, Tnc, Gpr88, Arhgap25), when deleted the animals display dendritic arborization phenotypes similar to the Nrg4-/- mice, or have been implicated in neurological disorders related to cortical development. At the very minimum, the authors should use anti-NRG4 or anti-ErbB4 antibodies to double immuno-label with anti-Fezf2 or anti-Gpr88, etc. (as all these antibodies are commercially available) in P0, P10 and P30 pyramidal neurons in the motor cortex. This will better link the role of Nrg4-ErbB4 signaling in cortical motor neuron development and strength the current study.

We appreciate this suggestion, and therefore carried double immune-labelling experiments using Fezf2 and Gpr88 together with NRG4 and ErbB4 in the motor cortex of postnatal day 10 and 30 mouse brains. The details of our observations are described in the text, validate the single-cell RNA sequencing data and strengthen this study and the requirement of NRG4 in dendritic outgrowth and overall size of pyramidal neurons.

2. The rotarod phenotype seemed very specific to the Nrg4-/- animals as other behavioral tests (elevated plus maze, open field and novel object recognition) showed that the mutants have normal anxiety levels and memory. However, from a previous study by Paramo et al. (2018), it was shown that both Nrg4 and ErbB4 mRNAs are also expressed in the cerebellum at P0. Nrg4 mRNA levels in the cerebellum are even higher than the cortex. Therefore, the motor learning deficits exhibited by the Nrg4-/- animals could be due to a contribution from the cerebellum. There is a conditional Nrg4 knockout mouse line available (Nugroho et al., 2018). It is noted that during this time there might be difficulty in obtaining mice from other labs or even repository. However, the authors should acknowledge this flaw in their analysis and modify the interpretation of their results to include the cerebellum as a likely contribution to the rotarod phenotype.

It is true that the levels of Nrg4 mRNA are higher in the cerebellum at P0. We have, however, explored whether Nrg4-/- Purkinje neurons display morphological defects and we did not observe any obvious and significant defect in the morphology of these neurons at P12. The use of a Nrg4-flox mouse line is a good suggestion as it would allow the study of Nrg4 specific functions for the development of different brain regions.

We have included this information in the discussion to suggest a potential role of the cerebellum in the motor phenotype we observed in the Nrg4 knockout mice.

3. The possible linkage between the reduced soma size and the motor defects is not clear and likely to be the result of reduced dendritic tree reported in previous paper.

It is likely that both the soma and dendrite phenotypes contribute to the motor defects. However, we cannot comment on the relative importance of each phenotypic change to the motor defects. We have modified the text accordingly to include this point.

4. The suggested autocrine model in which both the receptors and ligands are expressed by the same neurons is not well substantiated. This claim is based on a bioinformatics analysis of single cell RNA seq. In this analysis they find that about 75% of GABAergic neurons, mostly in L5, express Erbb4 while some of these cells also express Nrg4. This clearly does not prove their model. There was no attempt to support this model either by double staining of receptor and ligand in sections or even in vitro by using a blocking antibody (or other means of inhibition) to block Nrg4 in WT and test soma size.

In a previous paper, Paramo et al. reported double immuno-labeling of NRG4 and ErbbB4, NRG4 and Emx1 and ErbB4 and Emx1 in embryonic cortical neurons, and of NRG4 and MAP2 in the motor cortex of P10 mice. In addition, in order to validate the RNA seq data we have performed double immuno-labeling of NRG4 and Fez2f, NRG4 and Gpr88, ErbB4 and Fez2f, ErbB4 and Gpr88 and NRG4 and ErbB4 and found that Fezf2 and Gpr88--expressing neurons co-express ErbB4 and NRG4. Furthermore, we show that NRG4 neurons co-express ErbB4 at P10 and P30. Thus, it is likely that NRG4acts at least in part by an autocrine mechanism. However, we cannot exclude action by other mechanisms. We have included these new observations and modified the text accordingly.

Finally, here are a couple of minor concerns.

The quantifications in Fig. 3B-E shows statistical significance only between WT versus Nrg4-/- and Nrg4-/- versus Nrg4-/- + NRG4, was there statistical significance between WT versus Nrg4-/- + NRG4? If there is a full rescue of the soma size with the addition of exogenous NRG4, then no significance is expected, but if it is only a partial rescue then there could still be a significance between the two conditions. Statistical analysis between WT versus Nrg4-/- +NRG4 is needed.

The single cell data shown in Fig. 4 should be shown first, along with new double immuno-labelling results of Nrg4/ErbB4 with Fezf2 or Gpr88, etc. at early postnatal ages.

At 3 DIV the area of WT neurons versus Nrg4-/- neurons treated with NRG4 is not significantly different (mean 167.6 {plus minus} 4.04 μm2 vs 164.6 {plus minus} 2.94 μm2, respectively; adjusted p value 0.8106), nor is the perimeter (mean {plus minus} SEM: 49.93 {plus minus} 0.6 μm2 vs 49.65 {plus minus} 0.51 μm2, respectively, adjusted p value 0.9343,) which indicates full rescue by NRG4.

This effect is lost at 9 DIV, where the soma area of WT neurons versus Nrg4-/- neurons treated with NRG4 is significantly different (mean {plus minus} SEM: 258.3 {plus minus} 8.3 μm2 vs 233.1 {plus minus} 6.49 μm2, respectively; adjusted p value 0.0384), and so is the perimeter (mean {plus minus} SEM: 64.25 {plus minus} 1.31 μm vs 60.62 {plus minus} 0.98 μm, respectively; adjusted p value 0.0378) indicating that NRG4 only rescued the decrease in soma size partially. These details are now included in the text.

We included the new immuno-labelling results are shown in modified Figure 1.
